# Antibiotic overuse in the primary health care setting: a secondary data analysis of standardised patient studies from India, China and Kenya

**DOI:** 10.1136/bmjgh-2020-003393

**Published:** 2020-09-15

**Authors:** Giorgia Sulis, Benjamin Daniels, Ada Kwan, Sumanth Gandra, Amrita Daftary, Jishnu Das, Madhukar Pai

**Affiliations:** 1 Epidemiology, Biostatistics and Occupational Health, McGill University, Montreal, Québec, Canada; 2 McGill International TB Centre, McGill University, Montreal, Québec, Canada; 3 McCourt School of Public Policy, Georgetown University, Washington, District of Columbia, USA; 4 School of Public Health, University of California Berkeley, Berkeley, California, USA; 5 Division of Infectious Diseases, Department of Medicine, Washington University in Saint Louis, Saint Louis, Missouri, USA; 6 Dahdaleh Institute of Global Health Research, York University, Toronto, Ontario, Canada; 7 Centre for the AIDS Programme of Research in South Africa (CAPRISA), University of KwaZulu-Natal, Durban, KwaZulu-Natal, South Africa; 8 Centre for Policy Research, New Delhi, Delhi, India; 9 Manipal McGill Program for Infectious Diseases, Manipal Centre for Infectious Diseases, Manipal Academy of Higher Education, Manipal, Karnataka, India

**Keywords:** other study design, treatment, epidemiology

## Abstract

**Introduction:**

Determining whether antibiotic prescriptions are inappropriate requires knowledge of patients’ underlying conditions. In low-income and middle-income countries (LMICs), where misdiagnoses are frequent, this is challenging. Additionally, such details are often unavailable for prescription audits. Recent studies using standardised patients (SPs) offer a unique opportunity to generate unbiased prevalence estimates of antibiotic overuse, as the research design involves patients with predefined conditions.

**Methods:**

Secondary analyses of data from nine SP studies were performed to estimate the proportion of SP–provider interactions resulting in inappropriate antibiotic prescribing across primary care settings in three LMICs (China, India and Kenya). In all studies, SPs portrayed conditions for which antibiotics are unnecessary (watery diarrhoea, presumptive tuberculosis (TB), angina and asthma). We conducted descriptive analyses reporting overall prevalence of antibiotic overprescribing by healthcare sector, location, provider qualification and case. The WHO Access–Watch–Reserve framework was used to categorise antibiotics based on their potential for selecting resistance. As richer data were available from India, we examined factors associated with antibiotic overuse in that country through hierarchical Poisson models.

**Results:**

Across health facilities, antibiotics were given inappropriately in 2392/4798 (49.9%, 95% CI 40.8% to 54.5%) interactions in India, 83/166 (50.0%, 95% CI 42.2% to 57.8%) in Kenya and 259/899 (28.8%, 95% CI 17.8% to 50.8%) in China. Prevalence ratios of antibiotic overuse in India were significantly lower in urban versus rural areas (adjusted prevalence ratio (aPR) 0.70, 95% CI 0.52 to 0.96) and higher for qualified versus non-qualified providers (aPR 1.55, 95% CI 1.42 to 1.70), and for presumptive TB cases versus other conditions (aPR 1.19, 95% CI 1.07 to 1.33). Access antibiotics were predominantly used in Kenya (85%), but Watch antibiotics (mainly quinolones and cephalosporins) were highly prescribed in India (47.6%) and China (32.9%).

**Conclusion:**

Good-quality SP data indicate alarmingly high levels of antibiotic overprescription for key conditions across primary care settings in India, China and Kenya, with broad-spectrum agents being excessively used in India and China.

**Key questions**

**What is already known?**

- A recent systematic review and meta-analysis showed that, across 48 studies from 27 low-income and middle-income countries including China, India and Kenya, approximately half of all patients evaluated in outpatient primary care received an antibiotic prescription.
- Methods used to assess inappropriateness of antibiotic prescription, such as prescription audits, medical records and patient exit interviews, have multiple limitations.
- Standardised patients (SPs) offer a unique opportunity to explore prescribing practices and accurately estimate overprescription because case presentations are fixed by design, thus allowing comparisons across settings and providers.

**What are the new findings?**

- In this secondary analysis of data from nine SP studies carried out in India, Kenya and China, we provide a more unbiased prevalence estimate of antibiotic overprescription for selected clinical conditions (asthma, angina, watery diarrhoea, presumptive or confirmed tuberculosis (TB)) across a range of primary healthcare providers.
- About 30% of SP–provider interactions in China and 50% of those performed in India and Kenya resulted in inappropriate antibiotic prescription.
- Watch antibiotics (ie, broad-spectrum agents with higher potential for selecting resistance) were very commonly prescribed in India (about 50%) and China (over 32%), and some patients (0.8%) even received last-resort antibiotics belonging to the ‘Reserve’ group.
- In India, the average prevalence of antibiotic prescribing was 30% lower in urban versus rural areas, 55% higher among qualified providers compared with non-qualified ones and 19% higher for patients presenting with presumptive TB versus other conditions.

**What do the new findings imply?**

- Our findings indicate alarming levels of antibiotic overprescription for conditions that are frequently encountered in primary care, potentially leading to toxic effects and diagnostic delays.
- The choice of antibiotics given to patients is concerning, as several agents with high potential for resistance selection are often inappropriately prescribed.
- The SP methodology could prove useful to further investigate antibiotic prescribing practices and its underlying determinants, using other case presentations across a range of different contexts.

## Introduction

Antibiotic stewardship is critical for tackling antimicrobial resistance (AMR), especially in the context of the ongoing COVID-19 pandemic.^1^ In a recent systematic review on antibiotic prescription practices in primary care settings across low-income and middle-income countries (LMICs), we showed that approximately 50% of patients of any age seeking care for any reason received at least one antibiotic.^2^

However, determining inappropriate prescription in LMICs is a challenge, and a standardised tool for its assessment is currently unavailable. Inappropriate antibiotic prescribing can derive from a range of failings: (1) prescription in the absence of clinical indication (ie, ‘overprescription’), which not only produces zero benefit to the patient but can also be harmful (eg, drug toxicities or costs for patients); (2) failure to prescribe antibiotics when necessary (ie, ‘underprescription’); (3) suboptimal antibiotic choice with respect to aetiology (confirmed or presumptive), site, severity of infection and patient characteristics (eg, age, comorbidities and pregnancy status); (4) prescription of wrong dosage and/or duration of antibiotic treatment as compared with national and international guidelines.^3 4^

Methods used to assess inappropriateness, such as prescription audits, medical records and patient exit interviews, have multiple limitations.^3 5^ Electronic records are seldom available in LMICs, particularly in primary care, thus making accurate prescription audit tools difficult to implement. Also, the paucity and variation of clinical details that can be captured through medical records (paper-based or not), if they even exist, makes it even harder to determine the appropriateness of prescription.^3^ Patient exit interviews are commonly used alternatives but come with several major drawbacks that can result in poor and inaccurate estimates that are incomparable. Data collected in this manner are subject to recall bias, poor recall and limited clinical expertise among patients. Further, not only are clinical presentations highly heterogeneous but also the difficulty in actually determining what patients have makes comparisons very challenging for research.

A less biased method is the use of standardised patients (SPs), also known as ‘simulated’ or ‘mystery’ patients, that is, healthy individuals recruited from local communities and extensively trained to portray a standardised clinical condition to a healthcare provider.^5^ Since their clinical presentations are fixed by design, SPs offer an important opportunity to overcome the methodological limitations typical of other studies, thus making the assessment of inappropriateness of antibiotic use less biased and more accurate.^5^ Because the underlying illness is prespecified, the SP methodology allows accurate assessment if an antibiotic is inappropriately prescribed. The SP approach is not affected by poor recall, recall bias or the Hawthorne effect, which is commonly observed in patient exit interviews and direct observations of patient–provider encounters.^5^

Considering the aforementioned advantages, we performed a secondary analysis of prescription data from previously conducted SP studies in three LMICs (India, China and Kenya) with two objectives: (1) to estimate the overall proportion of SP–provider interactions (separately for pharmacy-based and health facility-based studies) that resulted in prescription or dispensing of at least one antibiotic in the absence of clinical indication (ie, overprescription) and (2) to identify factors associated with antibiotic overprescribing in health facilities.

## Methods

### Study design and data sources

Data on SP–provider interactions (ie, completed SP visits with a provider at a health facility or a pharmacy) from studies conducted by members of our team (India and Kenya) or had used SP cases developed by our team or obtained from publicly accessible sources (China) were gathered to compile a pooled dataset for secondary analyses.^6–15^ The methods used are described in our published manual and toolkit on how to conduct SP studies.^5^

Among studies carried out in India, four involved primary health facilities across five sites (Delhi, Mumbai, Patna, three districts in the State of Madhya Pradesh, and Birbhum district in the State of West Bengal),^6–9^ while two were performed in pharmacies located in four different areas (Mumbai, Patna, Delhi and Udupi district of Karnataka).^10 11^ We also examined data from a pilot study carried out in Nairobi (Kenya) and two studies completed in rural areas of China (Shaanxi, Sichuan and Anhui provinces), all involving only primary healthcare providers.^12–15^

Information regarding medications prescribed by healthcare providers were collected in these published SP studies but were not analysed in depth, especially with regard to inappropriate use. This is because, in most instances, the primary publications focused on overall quality of care, rather than the specific components of care.

### Provider selection in original studies

Sampling approaches adopted in each primary study from which our data were drawn are summarised in table 1. For the two pharmacy-based studies, a random sample of pharmacies was selected from a comprehensive list of all those eligible obtained from relevant authorities.^10 11^ In six of the other eight studies, healthcare providers were randomly sampled after performing a census or street-by-street mapping in the study areas.^7–9 13–15^ A convenience sample of practitioners was selected in two pilot studies respectively performed in Delhi and Nairobi.^6 12^ A waiver of provider consent was obtained in four out of nine studies, all carried out in India, two of which involved pharmacies.^7 9–11^ In all the others, verbal or written informed consent was sought at least 6 weeks prior to the commencement of SP–provider interactions in order to reduce the risk of SP detection. Yet, participation rates were very high (85%–100%) among eligible health practitioners, and non-participation was usually due to logistical issues on the day of the visits rather than active refusal to be involved in the project. Hence, it is reasonable to expect negligible differences between participants and non-participants, making non-response bias a minor concern. In all studies, SPs were randomly assigned to providers, and completion rates of SP–provider interactions were always very high.

**Table 1:** Main features of SP studies included in our analyses

Study site (year)	SP–provider interactions	Tracer conditions	Healthcare sector	Facility location	Provider selection approach	Provider consent	Provider participation*
China (2013)	600	Angina, child diarrhoea	Public	Rural	Census of all clinics designated under the New Cooperative Medical Scheme (ie, the major public health insurance programme in rural areas), followed by random selection of providers	Yes	100%
China (2015)	299	Presumptive TB	Public	Rural	Census of all public providers followed by random sampling from one prefecture in each of 3 provinces out of a total of 47 prefectures, chosen to be representative of rural health systems	Yes	274/274 (100%)
Kenya (2014)	166	Angina, asthma, child diarrhoea, presumptive TB	Public and private	Urban	Non-random convenience sample designed to include low-income, middle-income and high-income neighbourhoods in various Nairobi areas	Yes	46/49 (93.9%)
Madhya Pradesh, India (2010**–**2011)	1123	Angina, asthma, child diarrhoea	Public and private	Rural	Census of all medical care providers working in 60 villages randomly sampled in three districts in Madhya Pradesh; all public providers and qualified private providers were automatically sampled; for each public provider, the closest private practitioner was also sampled	No	Not applicable
Delhi, India (2014)	250	Presumptive and confirmed TB, presumptive MDR-TB	Private	Urban	Convenience sample (pilot study)	Yes	Not available
Mumbai and Patna, India (2014**–**2015)	2602	Presumptive and confirmed TB, presumptive MDR-TB	Private	Urban	Street-by-street mapping of private providers who were known to see adult outpatients with respiratory symptoms, followed by random sampling stratified by provider qualification and private provider interface agency registration status	No	Not applicable
Birbhum district, West Bengal, India (2012**–**2014)	823	Angina, respiratory distress, child diarrhoea	Private	Rural	Census of private health providers who had been practising for at least 3 years in 203 villages across Birbhum district	Yes	304/360 (84.4%)
Mumbai, Patna and Delhi, India (2014**–**2015)	1200	Presumptive TB, confirmed TB	Pharmacies	Urban	Convenience sample of 54 pharmacies from 28 low-income localities in Delhi (pilot phase), random sampling of pharmacies in Mumbai and Patna from a list of all pharmacies registered in the two cities	No	Not applicable
Udupi district, Karnataka, India (2018)	1522	For both adults and children: upper respiratory tract infection, diarrhoea, presumptive malaria	Pharmacies	Urban and rural	Of the 350 pharmacies registered in the district as per the local pharmacy association, 279 were considered eligible for the study after excluding those operating inside hospitals (47), those permanently closed or under renovations (10), those that could not be identified by the field team (4), those for veterinarian purposes only (1) and those used for SP training (10).	No	Not applicable

*For studies in which provider consent was required.

MDR-TB, multidrug resistant tuberculosis; SP, standardised patient; TB, tuberculosis.

### Tracer conditions

Tracer conditions (ie, SP case presentations) were defined similarly across SP studies, thus allowing comparisons across settings. Cases ranged from presumptive or confirmed tuberculosis (TB) (which requires specific anti-TB treatment as per WHO recommendations) to self-limiting infections, such as watery diarrhoea or upper respiratory tract illness (which only need support treatment, eg, rehydration therapy for diarrhoea), to non-communicable diseases like asthma or chest pain indicative of angina (these should be referred to a higher level of care). Importantly, none of such conditions requires antibiotics, which means that any antibiotic prescribed to SPs is deemed inappropriate by indication (ie, overprescription).

### Outcome assessment

Raw data from original studies were harmonised and recoded as needed. We used the available information on medications that were prescribed or dispensed during each SP–provider interaction to categorise individual drugs. Antibacterial agents were further classified using both the ATC (Anatomical–Therapeutic–Chemical) Index and the WHO Access–Watch–Reserve (AWaRe) framework.^16 17^ Fixed-dose combinations (FDCs) of antibiotics (eg, ciprofloxacin/ornidazole) were classified as ‘discouraged’ antibiotics as per WHO recommendations.

The primary outcome measure was expressed as the proportion of SP–provider interactions that resulted in antibiotic prescription or dispensing. Secondary outcomes were proportions of specific groups of antibiotics that were prescribed or dispensed both overall and across strata of key variables of interest. These proportions provide a direct measure of antibiotic overuse.

### Statistical analyses

For studies carried out in health facilities, we conducted country-level descriptive analyses and reported the crude proportion of SP–provider interactions that resulted in antibiotic prescription or dispensing. The overall proportion of prescribed or dispensed antibiotics, along with ATC-class and AWaRe group-specific proportions, was calculated across strata defined by key variables of interest, such as healthcare sector (public/private), facility location (urban/rural), provider qualification (qualified/non-qualified, defined based on whether they had at least a bachelor’s degree in medicine) and tracer conditions. For all prevalence proportions, we computed 95% CIs using bootstrapping in order to account for clustering at the study level.^18^

In order to examine the factors associated with antibiotic prescribing in health facilities in India, we fit a hierarchical Poisson regression model that allows direct estimation of adjusted prevalence ratios (aPRs) even if the outcome is common as in this case. Our model included a random intercept for studies and dummy variables for facility location, healthcare sector, provider qualification and tracer conditions as predictors.^19^ As we anticipated a fair amount of between-study heterogeneity, we decided to opt for a mixed model that could better account for it as compared with including the study or study site as a covariate. Among tracer conditions, only angina, asthma and presumptive TB could be included in order to avoid sparse data problems (ie, violations of the positivity assumption). The effect of all predictors was expected to be similar across studies, and therefore only fixed slopes were considered. These analyses were restricted to India because we had diverse and more data. We also considered alternative models and examined the pros and cons of each. A full description of our analyses is provided in online supplementary file 1.

10.1136/bmjgh-2020-003393.supp1 Supplementary data

Data from pharmacies were not pooled because contexts and tracer conditions were highly heterogeneous in the two available studies. Therefore, we only calculated prevalence proportions and 95% CIs of dispensed antibiotics, both overall and in stratified analyses.

All analyses were performed using Stata 16.

### Patient and public involvement

It was not possible to involve patients or the public in the design, conduct, reporting or dissemination plans of our research because this is a secondary analysis of previously conducted studies.

## Results

The main features of SP studies that were included in our analyses are summarised in table 1. A total of 4798 SP–provider interactions were completed in health facilities across urban and rural India, predominantly in the private sector. Both private and public healthcare providers were involved in the pilot study carried out in Nairobi (166 interactions), whereas studies from rural China only targeted the public sector (899 interactions). For these health facility-based studies, we first present summary statistics and then report results from our models.

### Antibiotic overuse across settings

In India, 2392 of 4798 (49.9%, 95% CI 40.8% to 54.5%) SP–provider interactions resulted in at least one antibiotic prescription (table 2). Similar proportions were observed in Nairobi (83 of 166; 50.0%, 95% CI 42.2% to 57.8%), while a lower percentage was found in the China studies (259 of 899; 28.8%, 95% CI 17.8% to 50.8%). However, in the latter case, the CI was substantially wide, reflecting the considerable between-study variance due to differences in tracer conditions evaluated.

**Table 2:** Number, proportion and bootstrapped 95% CIs (based on study-level clusters) of standardised patient–provider interactions in health facilities that resulted in prescription or dispensing of antibiotics across strata of key variables

Variable	Country							
	All		India		China		Kenya	
	n/N	Proportion (95% CI)	n/N	Proportion (95% CI)	n/N	Proportion (95% CI)	n/N	Proportion (95% CI)
At least one antibiotic	2734/5863	46.6 (33.4 to 53.9)	2392/4798	49.9 (40.8 to 54.5)	259/899	28.8 (17.8 to 50.8)	83/166	50.0 (42.2 to 57.8)
Antibiotics, n								
0	3129/5863	53.4 (46.1 to 66.6)	2406/4798	50.1 (45.4 to 57.9)	640/899	71.2 (49.2 to 71.2)	83/166	50.0 (42.2 to 57.8)
1	2465/5863	42.0 (31.4 to 47.4)	2159/4798	45.0 (39.8 to 48.2)	229/899	25.5 (25.5 to 42.8)	77/166	46.4 (39.2 to 54.2)
2	260/5863	4.4 (1.6 to 6.5)	225/4798	4.7 (1.4 to 6.6)	29/899	3.2 (3.2 to 7.7)	6/166	3.6 (1.2 to 6.6)
3	9/5863	0.2 (0.02 to 0.3)	8/4798	0.2 (0.03 to 0.3)	1/899	0.1 (0.1 to 0.3)	0/166	0
Health facility location								
Urban	1653/3018	54.8 (50.0 to 55.2)	1570/2852	55.0 (53.0 to 55.2)	–	–	83/166	50.0 (42.8 to 57.8)
Rural	1081/2845	38.0 (26.6 to 48.1)	822/1946	42.2 (39.0 to 46.7)	259/899	28.8 (17.8 to 50.8)	–	–
Healthcare sector								
Public	443/1321	33.5 (20.6 to 50.8)	156/367	42.5 (37.6 to 47.7)	259/899	28.8 (17.8 to 50.8)	28/55	50.9 (38.2 to 63.6)
Private	2291/4542	50.4 (40.8 to 54.5)	2236/4431	50.5 (50.2 to 54.5)	–	–	55/111	49.5 (40.1 to 51.6)
Provider qualification								
Qualified	1186/1906	62.2 (45.4 to 71.3)	1115/1768	63.1 (44.6 to 71.8)	71/138	51.4 (42.8 to 59.4)	NA	NA
Non-qualified	1358/3191	42.6 (38.7 to 48.6)	1277/3030	42.1 (37.8 to 47.9)	81/161	50.3 (42.9 to 57.8)	NA	NA
Clinical presentation								
Angina	169/955	17.7 (12.2 to 28.3)	115/598	19.2 (16.8 to 21.1)	29/315	9.2 (5.9 to 12.4)	25/42	59.5 (45.2 to 73.8)
Asthma	330/718	46.0 (44.0 to 50.2)	308/676	45.6 (43.5 to 49.0)	–	–	22/42	52.4 (38.1 to 66.7)
Child diarrhoea	490/997	49.1 (33.4 to 67.9)	399/672	59.4 (50.5 to 75.0)	78/285	27.4 (21.8 to 32.5)	13/40	32.5 (17.5 to 45.5)
Presumptive TB	1293/2253	57.4 (51.3 to 58.6)	1118/1912	58.5 (58.4 to 59.3)	152/299	50.8 (44.8 to 56.2)	23/42	54.8 (39.3 to 69.0)
Confirmed TB	194/404	48.0 (47.7 to 50.0)	194/404	48.0 (47.7 to 50.0)	–	–	–	–
Presumptive MDR-TB	258/536	48.1 (48.0 to 48.1)	258/536	48.1 (48.0 to 48.1)	–	–	–	–
Patient referred for further evaluation*								
Yes	101/767	13.2 (9.4 to 20.4)	65/498	13.1 (9.7 to 17.4)	33/263	12.5 (7.3 to 31.6)	3/6	50.0 (16.7 to 83.3)
No	2163/4384	50.7 (35.6 to 57.5)	1928/3628	53.1 (38.4 to 58.0)	226/636	35.5 (23.3 to 55.4)	67/120	55.8 (47.5 to 64.2)

*All child diarrhoea cases from India and Kenya (n=712) were excluded from this analysis because children were not directly assessed by the provider.

MDR-TB, multidrug resistant tuberculosis; NA, not available; TB, tuberculosis.

**Figure 1:**
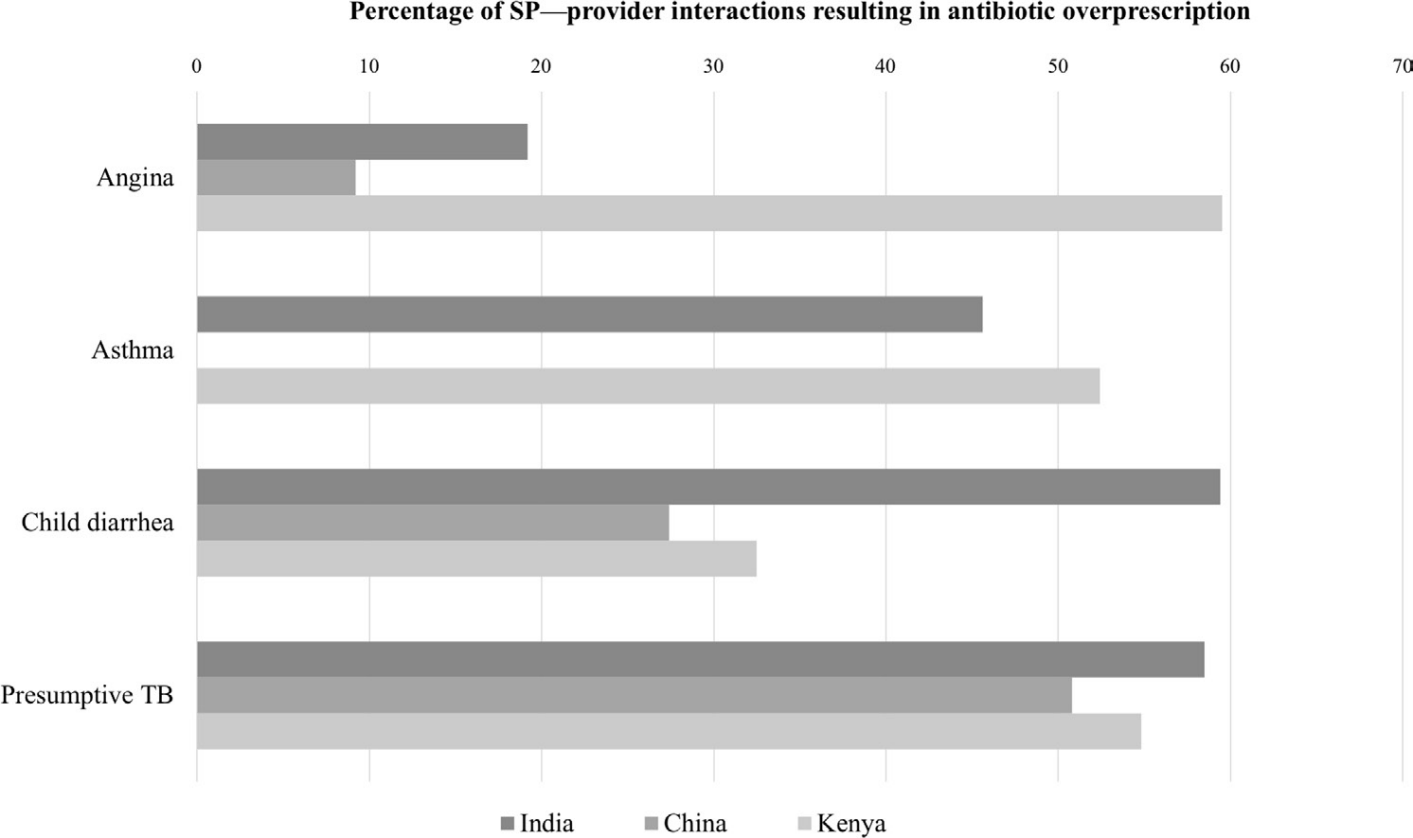
Crude percentage of SP—provider interactions resulting in antibiotic prescription/dispensing, by country and selected conditions (pharmacy-based studies are not included). SP, standardised patient; TB, tuberculosis.

In most instances, only one antibiotic was given during an individual SP–provider interaction; less than 5% of interactions across all settings resulted in two or more antibiotics prescriptions. Crude analyses of data from India indicate that antibiotic overprescription was more common among healthcare providers in urban areas, among those working in the private sector and among qualified professionals. Furthermore, antibiotics were largely overprescribed to patients presenting with a diverse range of clinical conditions in all countries (figure 1). In India, the percentage of subjects receiving antibiotics was close to 50% for most case types, with a peak of 59.4% (95% CI 50.5% to 75.0%) among child diarrhoea cases. However, for angina cases, it was 19.2% (95% CI 16.8% to 21.1%). About half of the visits for presumptive TB in China received antibiotics inappropriately, as opposed to 9.2% (95% CI 5.9% to 12.4%) of visits for suspicious angina and 27.4% (95% CI 21.8% to 32.5%) for child diarrhoea. Case-specific estimates from Nairobi are highly imprecise due to the small sample size.

### Type of antibiotics used

Across studies performed in India, 2768 antibiotics were given to 2392 patients. The top 10 most prescribed antibiotics across SP–provider interactions in India were azithromycin (381, 13.8%), amoxicillin+beta-lactamase inhibitor (344, 12.4%), amoxicillin (264, 9.5%), levofloxacin (202, 7.3%), cefixime (198, 7.2%), ofloxacin (165, 6.0%), ofloxacin+ornidazole (150, 5.4%), norfloxacin+tinidazole (136, 4.9%), ciprofloxacin (102, 3.7%) and cefpodoxime (88, 3.2%). Broad-spectrum agents with higher potential for selecting resistance (Watch antibiotics) were disproportionately represented (47.6%, 95% CI 26.8% to 54.0%), and even more so in urban areas (54.9%, 95% CI 54.9% to 55.4%) (table 3). This reflects the heavy use of quinolones, cephalosporins and macrolides that respectively accounted for 18.8% (95% CI 16.6% to 24.2%), 13.0% (95% CI 8.2% to 14.6%) and 15.4% (95% CI 4.1% to 19.3%) of all antibiotics prescribed in India. Nearly 80% of Watch antibiotics were given to SPs portraying a TB case (1086/1362). Three different last-resort or ‘Reserve’ antibiotics (colistin, linezolid and faropenem) were prescribed in a total of 23 SP–provider interactions in India, mainly for child diarrhoea (14/23).

**Table 3:** Frequency of antibiotics prescribed/dispensed in health facilities across study countries, overall and according to both the AWaRe and ATC classifications

Drug type	India						China	
	All settings		Urban India		Rural India			
	N	Proportion (95% CI)	N	Proportion (95% CI)	N	Proportion (95% CI)	N	Proportion (95% CI)
Any antibiotic	2768	–	1896	–	872	–	301	–
AWaRe classification								
Access	876	31.6 (30.0 to 38.9)	584	30.8 (29.8 to 30.8)	292	33.5 (29.9 to 37.1)	126	41.9 (36.2 to 47.2)
Watch	1317	47.6 (26.8 to 54.0)	1041	54.9 (54.9 to 55.4)	276	31.7 (21.2 to 40.3)	99	32.9 (27.6 to 37.9)
Reserve	23	0.8 (0.5 to 1.8)	8	0.4 (0.4 to 0.5)	15	1.7 (1.0 to 2.1)	1	0.3 (0.3 to 1.3)
Discouraged	334	12.1 (4.3 to 36.3)	50	2.6 (2.6 to 2.8)	284	32.6 (25.1 to 44.8)	1	0.3 (0.3 to 1.3)
Not available*	218	7.9 (5.4 to 10.8)	213	11.2 (11.2 to 11.5)	5	0.57 (0.3 to 1.0)	74	24.6 (19.9 to 29.2)
ATC classification								
Penicillin	711	25.7 (18.8 to 27.0)	535	28.2 (27.7 to 28.2)	176	20.2 (17.6 to 21.7)	68	22.6 (17.6 to 27.2)
Cephalosporin	361	13.0 (8.2 to 14.6)	294	15.0 (14.9 to 15.0)	76	8.7 (7.8 to 10.7)	75	24.9 (20.9 to 29.2)
First generation	21	0.8 (0.6 to 1.8)	9	0.5 (0.47 to 0.51)	12	1.4 (1.1 to 2.1)	0	0
Second generation	22	0.8 (0.2 to 1.1)	20	1.1 (1.1 to 1.2)	2	0.2 (0.2 to 0.4)	7	2.3 (0.7 to 4.0)
Third generation	318	11.5 (7.1 to 12.9)	256	13.5 (13.3 to 13.5)	62	7.1 (6.4 to 8.1)	1	0.3 (0.3 to 1.0)
Not available*	0	0	0	0	0	0	67	22.3 (18.3 to 26.6)
Macrolide	425	15.4 (4.1 to 19.3)	389	20.5 (20.4 to 21.3)	36	4.1 (4.1 to 4.3)	60	19.9 (15.6 to 24.3)
Quinolone	520	18.8 (16.6 to 24.2)	354	18.7 (18.5 to 18.7)	166	19.0 (18.5 to 26.8)	37	12.3 (9.0 to 15.9)
Tetracycline	67	2.4 (1.7 to 4.6)	34	1.8 (1.4 to 1.8)	33	3.8 (3.0 to 4.1)	0	0
Imidazole†	61	2.2 (0.8 to 7.1)	1	0.05 (0.05 to 0.06)	60	6.9 (6.3 to 7.5)	1	0.3 (0.3 to 1.3)
Sulfonamide‡	18	0.7 (0.2 to 1.9)	3	0.16 (0.16 to 0.17)	15	1.7 (0.9 to 2.1)	9	3.0 (1.3 to 5.0)
Aminoglycoside	6	0.2 (0.1 to 1.0)	0	0	6	0.7 (0.7 to 1.3)	45	15.0 (11.3 to 18.6)
Combinations§	289	12.1 (5.1 to 34.2)	50	2.6 (2.6 to 2.8)	284	32.6 (25.1 to 34.2)	1	0.3 (0.3 to 1.3)
Antimycobacterial	229	8.3 (0.3 to 10.9)	226	11.9 (11.9 to 12.2)	3	0.3 (0.2 to 0.5)	1	0.3 (0.3 to 1.3)
Other antibiotics	36	1.3 (1.0 to 2.4)	19	1.0 (0.1 to 1.0)	17	1.9 (1.8 to 2.6)	4	1.3 (0.3 to 2.7)

The unit of analysis is the individual drug, not the standardised patient–provider interaction.

*For these drugs, only the antibiotic class (eg, cephalosporin) was available.

†Only metronidazole was prescribed/dispensed.

‡Only trimethoprim–sulfamethoxazole was prescribed/dispensed.

§This category does not include combinations of antimycobacterial drugs.

ATC, Anatomical–Therapeutic–Chemical; AWaRe, Access–Watch–Reserve.

Discouraged antibiotics, that is, FDCs other than antimycobacterial drugs (such as norfloxacin+tinidazole or ofloxacin+ornidazole) accounted for 12.1%, of which all but one were given for child diarrhoea. Anti-TB medications represented 8.3% of antibiotics in India; almost all of them were given by healthcare providers in urban areas; and none could be considered appropriate based on the expected correct management of such cases.

About one-quarter of drugs prescribed in studies from China could not be categorised based on the AWaRe framework because only the drug class was reported. These were mainly cephalosporins, most likely second or higher generation, and therefore the overall proportion of Watch-group antibiotics is expected to be greater than 32.9% (table 3). Undefined cephalosporins were by far the most prescribed antibiotics in China (76/301, 25.2%), followed by gentamicin (45/301, 15.0%), amoxicillin (37/301, 12.3%), erythromycin (26/301, 8.6%) and levofloxacin (18/301, 6.0%).

Subgroup analyses of antibiotic prescription patterns among SP–provider interactions that took place in Nairobi were limited by the small sample size. However, 85.4% (76/89) of all antibiotics prescribed were first-line and narrow-spectrum agents from the ‘Access’ group, while the remaining belonged to the ‘Watch’ group.

### Factors associated with antibiotic overuse in India

**Figure 2:**
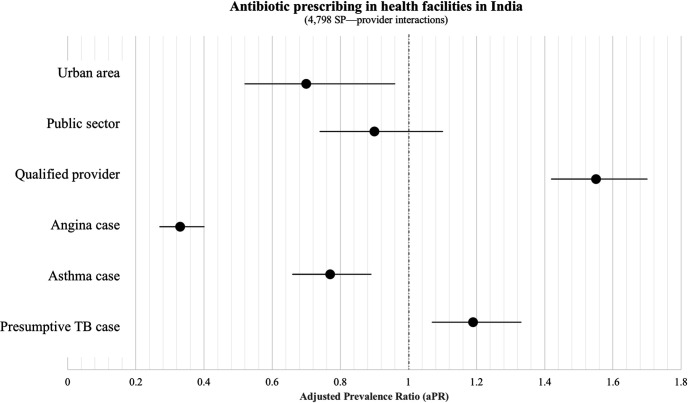
Factors associated with antibiotic prescribing/dispensing in health facilities in India. Covariate-adjusted prevalence ratios and their 95% CIs estimated from a hierarchical Poisson model are reported. SP, standardised patient; TB, tuberculosis.

Prevalence ratios of antibiotic overuse and their 95% CIs estimated through mixed-effects Poisson regression analysis are reported in figure 2. The adjusted prevalence of antibiotic prescribing was lower in urban versus rural areas (aPR=0.70; 95% CI: 0.52 to 0.96), for subjects presenting with suspicious angina (aPR=0.33; 95% CI: 0.27 to 0.40), and asthma (aPR=0.77; 95% CI: 0.66 to 0.89). Patients with presumptive TB were more likely to receive inappropriate antibiotics (aPR=1.19; 95% CI: 1.07 to 1.33) as compared with individuals with other clinical conditions. Qualified practitioners were more likely to prescribe antibiotics than non-qualified ones (aPR 1.55; 95% CI: 1.42 to 1.70).

The hierarchical Poisson model did not show any significant difference between public and private providers, but this is in contrast with what emerged from alternative models as described in online supplementary file 1.

### Antibiotic dispensing in pharmacies

Our secondary analysis of data from two pharmacy-based SP studies showed that over-the-counter antibiotic dispensing is also a common problem in various parts of India (table 4).

**Table 4:** Antibiotic dispensing in Indian pharmacies

Variable	Study setting			
	Udupi district, Karnataka (n=1522)		Mumbai, Delhi and Patna (n=1200)	
	n/N	Proportion (95% CI)	n/N	Proportion (95% CI)
Number of antibiotics				
1	55/1522	3.6 (2.6 to 4.6)	294/1,00	24.5 (22.2 to 27.0)
2	0	0	25/1200	2.1 (1.3 to 2.9)
Pharmacy location				
Urban	25/744	3.3 (2.2 to 4.7)	319/1200	26.6 (24.2 to 29.2)
Rural	30/778	3.9 (2.7 to 5.2)	–	–
Clinical presentation				
Adult with URI	11/250	4.4 (2.0 to 7.2)	–	–
Adult with diarrhoea	12/259	4.6 (2.3 to 7.1)	–	–
Adult with fever (malaria suspect)	10/252	4.0 (1.6 to 6.3)	–	–
Child with URI	0/252	0	–	–
Child with diarrhoea	20/250	8.0 (4.8 to 11.2)	–	–
Child with fever (malaria suspect)	2/259	0.8 (0.4 to 1.9)	–	–
Adult with presumptive TB	–	–	221/599	36.9 (33.1 to 40.7)
Adult with confirmed TB	–	–	98/601	16.3 (13.5 to 19.3)
Patient referred to health provider				
Yes	15/710	2.1 (1.1; 3.1)	41/497	8.2 (5.8; 10.9)
No	40/812	4.9 (3.6; 6.4)	278/703	39.5 (36.1; 43.2)

TB, tuberculosis; URI, upper respiratory illness.

In Udupi district (Karnataka state) the proportion of SP—pharmacist interactions that resulted in antibiotic dispensing was 3.6% (95% CI: 2.6 to 4.6), with a similar pattern in both urban and rural areas. In contrast, at least one antibiotic was dispensed in 319/1,200 interactions performed across Delhi, Mumbai and Patna, corresponding to 26.6% (95% CI: 24.2 to 29.2) of the total. However, a direct comparison between these two studies is not possible owing to the very different contexts involved and particularly to the different types of cases that were examined. As observed in studies from healthcare facilities, subjects presenting to pharmacies with symptoms suggestive of TB were generally more likely to receive an antibiotic as compared with other conditions.

The average proportion of Watch-antibiotics (predominantly quinolones and cephalosporins) dispensed across the three cities was 49.4% (95% CI: 43.9 to 54.4), ranging from 24.0% (95% CI: 15.0 to 32.0) in Mumbai to 60.9% (95% CI: 55.1 to 67.1) in Patna. A deeper evaluation of antibiotic dispensing in Udupi district is limited by the small sample size. Only 55 antibiotics were dispensed across 1522 interactions, thus making subgroup analyses less meaningful. Yet, it is worth highlighting that nearly half of these antibiotics were discouraged FDCs of two antibiotics, whereas the remaining were almost equally distributed among Access- and Watch-groups. More details regarding the types of antibiotics dispensed across pharmacies in both studies are presented in online supplementary file 2.

10.1136/bmjgh-2020-003393.supp2 Supplementary data

## Discussion

Our analysis of past SP studies involving 4798 SP–provider interactions in India showed that healthcare providers in primary care settings prescribed antibiotics to about half (49.9%) of patients presenting with clinical conditions that do not require antibiotics. Antibiotic overprescribing was found to be similar (50% of SP–provider interactions) in a small SP study carried out in Nairobi, Kenya. Pooled data from two studies conducted in China showed lower levels of antibiotic overuse (28.8%), but it should be noted that percentages differed substantially across individual studies, likely reflecting the different type of cases being involved. In fact, SP–provider interactions involving presumptive TB cases were more likely to result in antibiotic prescription as compared with other clinical conditions. Among the two pharmacy-based SP studies done in India,^10 11^ the proportion of antibiotic dispensing was 26.6% and 3.6%, respectively.

Although our focus was on LMICs, the overuse of antibiotics is not confined to LMICs. Large population-based cohort data have shown that antibiotic overuse in ambulatory settings across the United States was 30% among children and 17% among adults with certain respiratory tract illnesses for which antibiotics are not indicated (eg, asthma, allergies, acute bronchitis or bronchiolitis).^20^ An analysis of antibiotic prescription practices based on administrative data from Ontario, Canada, recently reported an overall rate of unnecessary antibiotic prescribing in primary care of 15.4%, though much higher percentages were observed for some respiratory conditions such as acute bronchitis (52.6%).^21^ However, a direct comparison with higher income countries cannot be done due to differences in study methodologies and local epidemiology.

Nearly 50% of all antibiotics prescribed in the context of India SP studies belonged to the ‘Watch’ list, with a peak of 80% among patients presenting with symptoms suggestive of TB, which is consistent with national antibiotic sales.^22^ Watch-antibiotics accounted for almost 33% of all antibiotics across China SP studies, but this is likely underestimated because nearly one quarter of all antibiotics could not be classified due to insufficient information. Of note, we observed a large use of cephalosporins (presumably second or third generation ones), which is in line with previous findings from drug sales analyses and prescription audits conducted in various parts of China.^2 23 24^ In contrast, the small SP study conducted in Nairobi revealed that over 85% of prescribed antibiotics were from the ‘Access’ group, and half of these were either trimethoprim/sulfamethoxazole or amoxicillin. This is in line with that observed in another SP study carried out in urban public primary healthcare facilities in South Africa, where 10/119 (8.4%) interactions for presumptive TB resulted in antibiotic prescriptions, all of which belonged to the access group.^25^ As with the Nairobi study, however, the small sample size does not allow to draw meaningful conclusions on antibiotic prescribing patterns in the area.

Discouraged FDCs of antibiotics were commonly given in India but not in other settings, accounting for 10.4% of the total. FDCs were finally banned in India in September 2018, thus leaving hope for a change in the near future.

Alarmingly, we observed the use of some ‘Reserve’ antibiotics in primary care settings. In India, oral colistin was prescribed for paediatric diarrhoea, and faropenem was given to one patient with presumptive TB. This is very concerning as parenteral colistin is the last resort drug for treatment of extremely drug-resistant Gram-negative infections,^26^ and using the oral formulation could drive resistance in the community. Similarly, faropenem is an oral penem antibiotic which has been shown to cause cross-resistance to intravenous carbapenems.^27^ In China SP studies, one presumptive TB case received aztreonam, indicated for treatment of serious infections due to drug-resistant Gram-negative bacteria.

According to our findings from India, antibiotic overuse was particularly common in rural areas, among qualified providers and for patients presenting with presumptive TB. Besides leading to potentially dangerous diagnostic delays,^28 29^ the unnecessary use of antibiotics causes harms to the patient in terms of drug-associated adverse events and increased out-of-pocket costs.

While normative boundaries may partly explain why qualified providers prescribed more antibiotics than non-qualified ones as observed in our analyses for India, the widespread overuse of antibiotics suggests that important training gaps likely exist. However, prescribing behaviours among healthcare providers also depend on a number of other factors, including financial incentives from pharmaceutical companies, patient expectations and requests, or just old habits that are hard to die.^8 30 31^

The biggest strength of our study lies in the nature and quality of the data used to investigate the extent and patterns of antibiotic overprescribing. Although previous research had already highlighted that Watch group antibiotics are highly prescribed across India and China, such studies could not provide a clear picture of inappropriate antibiotic use owing to the limited amount of clinical information available from prescription audits and evaluations of drug sales data.^32–34^ Among the main advantages of using SPs to evaluate prescription practices is the fact that tracer conditions are standardised.^5^ In all studies included in our analyses, such conditions were very common illnesses that are frequently encountered in primary care and that require a well-defined diagnostic and therapeutic management that does not involve antibiotic use.

Furthermore, representative samples of healthcare providers from public and/or private sectors were selected in all SP studies conducted in India, with the only exception of one relatively small pilot study in Delhi. In this pooled dataset, private practitioners were much more represented than public providers, but we lacked statistical power to make appropriate comparisons between the two groups. Yet, this distribution well reflects the fact that about 75% of outpatient visits in India take place in the private sector, with nearly 70% of primary care in the country being delivered by informal providers.^35 36^

Of note, available data originated from a range of geographical areas with different sociocultural and economic profiles and could be generalisable to similar contexts in India. For all these reasons, the representativeness of our findings is very good, and selection bias is likely negligible due to the robust mapping and sampling approach used across all SP studies.

There are limitations in our study. First, the SP study data from China and Kenya were limited and lacked generalisability. Second, our analyses were restricted to overprescription and to a limited number of clinical scenarios. Third, we could not investigate other important forms of inappropriate antibiotic use, such as the choice of the incorrect drug and dosage to treat a given infection. This is an intrinsic limitation that arises from the type of tracer conditions used across SP studies so far. Although the SP methodology was initially implemented to assess overall quality of care in LMICs and to evaluate educational/behavioural programmes in high-income countries, this approach is being increasingly adopted to gain insight into medication use, and especially drug dispensing practices among pharmacists. Data recording systems in SP studies are therefore improving in order to facilitate the collection of key details regarding medications that were harder to capture from studies whose main objective was not related to drug use.

In conclusion, the prevalence of antibiotic overprescribing estimated from SP studies ranged from 29% in China to 50% in India and Kenya, and Watch antibiotics accounted for a large proportion of antibiotics prescribed in both India and China. Combining the SP methodology with new tracer conditions would allow overcoming many of the typical limitations of most studies aimed at evaluating inappropriate antibiotic use in greater detail. SPs represent a unique opportunity to further explore prescription practices among healthcare providers, including the management of common infectious diseases, such as pneumonia or urinary tract infections, that contribute substantially to the overall antibiotic use in primary care. Future studies also need to focus on untangling the channels for antibiotic overprescription and better understand the determinants of such practice among public and private healthcare providers in various contexts.

The extent of antibiotic overuse in primary care across LMICs is a serious concern and requires targeted antimicrobial stewardship interventions aimed at improving rational and locally adapted prescribing practices. An active involvement of private providers in all such interventions would be essential to ensure uptake, particularly in countries where the private sector plays a major role in healthcare. Greater efforts are also necessary to develop and scale up accurate point-of-care tests that could guide therapeutic choices where resources are scarce. Additional research is also required to evaluate whether antibiotic use (especially use of drugs such as azithromycin and hydroxychloroquine) will dramatically increase as a consequence of the COVID-19 pandemic, and concerns have already been raised about the implications for AMR.^37^
