# Supplementary material for: Antibiotic overuse in the primary health care setting: a secondary data analysis of standardised patient studies from India, China and Kenya
Source: BMJ Glob Health. 2020 Sep 15;5(9):e003393. doi: 10.1136/bmjgh-2020-003393 (PMC7493125; doi:10.1136/bmjgh-2020-003393)
Supplement: Supplementary data [file bmjgh-2020-003393supp001.pdf]

Supplementary File 1

Evaluation of factors associated with antibiotic prescribing in India – description of all analyses

To examine the factors associated with antibiotic prescribing or dispensing in India, we conducted univariate and multivariate analyses, where the outcome of interest was a binary variable for antibiotic prescription. Four different models were utilized to directly estimate prevalence ratios (PRs) and results were compared to evaluate consistency and identify the most appropriate analytical approach.[1-4]

We first fitted a Poisson regression model with dummy variables for facility location, healthcare sector, provider qualification, and tracer condition as predictors (Model 1). Because the high frequency of the outcome generates issues of under-dispersion, robust variance estimates were obtained. This model was expected to perform fairly well in this context because the sample size is large.[4,5] Among tracer conditions, only angina, asthma and presumptive TB could be included in order to avoid positivity violations.

Secondly, we used log-binomial regression including the same predictors as before, with cluster-based robust variance estimator (Model 2). Thirdly, a hierarchical Poisson regression model with a random intercept for studies was utilized (Model 3).

Lastly, a hierarchical logistic regression model, also including a random intercept for studies, was fitted (Model 4). In Models 3 and 4, the effect of predictors was expected to be similar across studies, and therefore only fixed slopes were considered. Exponentiated estimates from simpler versions of the models described above, including up to two predictor variables, were checked against Mantel-Haenszel-adjusted PRs to evaluate the models' performance.[4]

Prevalence ratios of antibiotic overuse and their 95% CIs estimated through univariable and multivariable analyses for Models 1-4 are reported in Table A. All models were concordant in showing that the adjusted prevalence of antibiotic prescribing/dispensing was lower in urban versus rural areas, for subjects presenting with suspicious angina, and for those presenting with asthma. Adjusted prevalence ratios were consistently greater than one for patients with presumptive TB, suggesting that this condition more often leads providers to inappropriately

prescribe antibiotics. Qualified practitioners also appear to be more likely to prescribe antibiotics than non-qualified ones.

Our results are inconclusive with regards to the healthcare sector (public versus private). It should be noted that the distribution of private and public providers in the sample population was substantially uneven, leading to insufficient statistical power to detect meaningful differences between the two groups. In fact, private practitioners constitute the largest majority of all providers involved across studies. The results of a subgroup analysis of antibiotic prescribing among private practitioners are presented in Table B.

Furthermore, a head-to-head comparison of public versus private sector was only undertaken in rural Madhya Pradesh, where SPs only presented cases of angina, asthma and child diarrhea. Hence, we conducted subgroup analyses restricted to this study. Both log-binomial and robust Poisson did not identify any statistically significant differences between public and private practitioners in the selected area

As expected, the hierarchical logistic regression model substantially overestimated the prevalence ratios owing to the high frequency of the outcome under investigation.[6] Estimates from simpler versions of all models except for the hierarchical logistic one, were perfectly aligned with those obtained with Mantel-Haenszel method. However, we observed lack of convergence with the full log-binomial regression model, as is often the case with this approach.[3] Robust Poisson and hierarchical Poisson with a random intercept for studies yielded pretty similar estimates. Unsurprisingly, the hierarchical model produced larger standard errors because it accounts for both between- and within-study variance.[1]

References:

1. Stewart GB, Altman DG, Askie LM, Duley L, Simmonds MC, Stewart LA. Statistical analysis of individual participant data meta-analyses: a comparison of methods and recommendations for practice. *PLoS One* 2012; **7**(10): e46042.

2. Skov T, Deddens J, Petersen MR, Endahl L. Prevalence proportion ratios: estimation and hypothesis testing. *Int J Epidemiol* 1998; **27**(1): 91-5.

3. Petersen MR, Deddens JA. A comparison of two methods for estimating prevalence ratios. *BMC Med Res Methodol* 2008; **8**: 9.

4. Barros AJ, Hirakata VN. Alternatives for logistic regression in cross-sectional studies: an empirical comparison of models that directly estimate the prevalence ratio. *BMC Med Res Methodol* 2003; **3**: 21.

5. Zou G. A modified poisson regression approach to prospective studies with binary data. *Am J Epidemiol* 2004; **159**(7): 702-6.

6. Thompson ML, Myers JE, Kriebel D. Prevalence odds ratio or prevalence ratio in the analysis of cross sectional data: what is to be done? *Occup Environ Med* 1998; **55**(4): 272-7.

Sulis G, *et al. BMJ Global Health* 2020; 5:e003393. doi: 10.1136/bmjgh-2020-003393

Table A Factors associated with antibiotic prescribing/dispensing in India: results of univariate and multivariate analyses using four different models based on 4,798 SP-provider interactions.

Predictor	**Model 1** (Poisson with robust variance estimates)		**Model 2** (Log-binomial with clustering)		**Model 3** (Poisson with random intercept)		**Model 4** (Logistic with random intercept)	
	**Univariable**	**Multivariable**	**Univariable**	**Multivariable**	**Univariable**	**Multivariable**	**Univariable**	**Multivariable**
	PR (95% CI)	*PR (95% CI)*	*PR (95% CI)*	*PR (95% CI)*	*PR (95% CI)*	*PR (95% CI)*	*OR (95% CI)*	*OR (95% CI)*
**Urban areas**	1.30 (1.23 – 1.39)	0.74 (0.67 – 0.81)	1.30 (1.16 – 1.47)	0.53 (0.53 – 0.54)	1.30 (1.20 – 1.42)	0.70 (0.52 – 0.96)	1.61 (1.29 – 2.00)	0.47 (0.26 – 0.86)
**Public sector**	0.84 (0.75 – 0.95)	0.77 (0.68 – 0.89)	0.84 (0.72 – 0.98)	0.62 (0.62 – 0.63)	1.09 (0.90 – 1.33)	0.90 (0.74 – 1.10)	1.20 (0.93 – 1.55)	0.77 (0.58 – 1.02)
**Qualified provider**	1.50 (1.42 – 1.58)	1.47 (1.39 – 1.55)	1.50 (1.17 – 1.91)	1.18 (indeterminate)	1.57 (1.44 – 1.72)	1.55 (1.42 – 1.70)	2.66 (2.33 – 3.03)	2.71 (2.36 – 3.11)
**Conditions**								
**Angina**	0.35 (0.30 – 0.42)	0.33 (0.28 – 0.40)	0.35 (0.32 – 0.40)	0.23 (0.21 – 0.25)	0.36 (0.30 – 0.44)	0.33 (0.27 – 0.40)	0.21 (0.17 – 0.26)	0.16 (0.12 – 0.21)
**Asthma**	0.90 (0.83 – 0.98)	0.77 (0.69 – 0.85)	0.90 (0.78 – 1.04)	0.54 (0.51 – 0.57)	1.10 (0.95 – 1.27)	0.77 (0.66 – 0.89)	1.21 (1.00 – 1.47)	0.56 (0.45 – 0.70)
**Presum. TB**	1.32 (1.25 – 1.40)	1.19 (1.11 – 1.29)	1.32 (1.18 – 1.49)	1.21 (1.20 – 1.22)	1.26 (1.13 – 1.40)	1.19 (1.07 – 1.33)	1.58 (1.35 – 1.84)	1.50 (1.27 – 1.76)

Abbreviations: CI, confidence interval; OR, odds ratio; PR, prevalence ratio; TB, tuberculosis

Sulis G, *et al. BMJ Global Health* 2020; 5:e003393. doi: 10.1136/bmjgh-2020-003393

Table B Factors associated with antibiotic prescribing/dispensing among private providers in India: results of univariate and multivariate subgroup analyses using hierarchical Poisson models based on 4,431 SP-provider interactions.

**Predictor**	**Univariable analysis**	**Multivariable analysis**
	PR (95% CI)	*PR (95% CI)*
**Urban areas**	1.29 (1.14 – 1.47)	0.67 (0.50 – 0.91)
**Qualified provider**	1.58 (1.44 – 1.73)	1.54 (1.41 – 1.69)
**Conditions**		
**Angina**	0.33 (0.27 – 0.43)	0.29 (0.23 – 0.36)
**Asthma**	1.03 (0.88 – 1.21)	0.70 (0.59 – 0.83)
**Presumptive TB**	1.25 (1.12 – 1.39)	1.19 (0.50 – 0.74)

Abbreviations: CI, confidence interval, PR, prevalence ratio; TB, tuberculosis
