# Supplementary material for: Antibiotic overuse in the primary health care setting: a secondary data analysis of standardised patient studies from India, China and Kenya
Source: BMJ Glob Health. 2020 Sep 15;5(9):e003393. doi: 10.1136/bmjgh-2020-003393 (PMC7493125; doi:10.1136/bmjgh-2020-003393)
Supplement: Supplementary data [file bmjgh-2020-003393supp002.pdf]

Supplementary File 2

Frequency of antibiotics dispensed in pharmacies, overall and according to both the AWaRe (Access – Watch – Reserve) and ATC (Anatomical – Therapeutic – Chemical) classifications.

Drug type	Udupi district, Karnataka		Mumbai, Delhi and Patna	
	**N**	**Proportion (95% CI)**	**N**	**Proportion (95% CI)**
**Any antibiotic**	55	-	344	-
**AWaRe Classification**				
Access	16	29.1 (16.4; 40.0)	172	50.0 (45.1; 55.2)
Watch	12	21.8 (12.7; 32.7)	170	49.4 (43.9; 54.4)
Reserve	0	-	0	-
Discouraged	25	45.5 (32.7; 58.2)	2	0.6 (0.3; 1.5)
Other*	2	3.6 (1.8; 9.1)	0	-
**ATC Classification**				
Penicillin	11	20.0 (9.1; 30.9)	163	47.4 (41.9; 52.3)
Cephalosporin	5	9.1 (3.6; 16.4)	38	11.0 (7.8; 14.5)
Macrolide	1	1.8 (1.8; 7.3)	57	16.6 (12.5; 20.6)
Quinolone	6	10.9 (3.6; 20.0)	81	23.5 (18.6; 28.2)
Tetracycline	0	-	2	0.6 (0.3; 1.5)
Imidazole†	5	9.1 (1.8; 18.2)	0	-
Combinations#	25	45.5 (32.7; 58.2)	2	0.6 (0.3; 1.5)
Other antibiotics§	2	3.6 (1.8; 9.1)	1	0.3 (0.29; 0.9)

Note: The unit of analysis is the individual drug, not the SP-provider interaction.
* In the Udupi study, antibiotics classified as “other” were all quiniodochlor.
† Only metronidazole was dispensed.
\# This category does not include combinations of anti-mycobacterial drugs.
§ Quiniodochlor in the Udupi study and chloramphenicol in the other study.
